# Osteoprotegerin CGA Haplotype Protection against Cerebrovascular Complications in Anti-CCP Negative Patients with Rheumatoid Arthritis

**DOI:** 10.1371/journal.pone.0106823

**Published:** 2014-09-03

**Authors:** Fernanda Genre, Raquel López-Mejías, Mercedes García-Bermúdez, Santos Castañeda, Carlos González-Juanatey, Javier Llorca, Alfonso Corrales, Begoña Ubilla, José A. Miranda-Filloy, Trinitario Pina, Carmen Gómez-Vaquero, Luis Rodríguez-Rodríguez, Benjamín Fernández-Gutiérrez, Alejandro Balsa, Dora Pascual-Salcedo, Francisco J. López-Longo, Patricia Carreira, Ricardo Blanco, Isidoro González-Álvaro, Javier Martín, Miguel A. González-Gay

**Affiliations:** 1 Epidemiology, Genetics and Atherosclerosis Research Group on Systemic Inflammatory Diseases, Rheumatology Division, IDIVAL, Santander, Spain; 2 Instituto de Parasitología y Biomedicina López-Neyra, IPBLN-CSIC, Granada, Spain; 3 Department of Rheumatology, Hospital Universitario la Princesa, IIS-Princesa, Madrid, Spain; 4 Cardiology Division, Hospital Universitario Lucus Augusti, Lugo, Spain; 5 Department of Epidemiology and Computational Biology, School of Medicine, University of Cantabria, and CIBER Epidemiología y Salud Pública (CIBERESP), IDIVAL, Santander, Spain; 6 Division of Rheumatology, Hospital Universitario Lucus Augusti, Lugo, Spain; 7 Department of Rheumatology, Hospital Universitario Bellvitge, Barcelona, Spain; 8 Department of Rheumatology, Hospital Clínico San Carlos, Madrid, Spain; 9 Department of Rheumatology, Hospital Universitario La Paz, Madrid, Spain; 10 Department of Rheumatology, Hospital General Universitario Gregorio Marañón, Madrid, Spain; 11 Department of Rheumatology, Hospital Universitario 12 de Octubre, Madrid, Spain; Centro de Investigación Príncipe Felipe – CIPF, Spain

## Abstract

**Introduction:**

Rheumatoid arthritis is an inflammatory disease with high incidence of cardiovascular disease due to accelerated atherosclerosis. Osteoprotegerin (OPG) has been associated with increased risk of atherosclerotic disease in the general population. Several polymorphisms in the *OPG* gene with functional effects on cardiovascular disease in non-rheumatic individuals have been described. Therefore, we aimed to analyze the effect of three of these functional *OPG* polymorphisms on the risk of cardiovascular disease in a large and well-characterized cohort of Spanish patients with rheumatoid arthritis.

**Methods:**

Three *OPG* gene variants (rs3134063, rs2073618 and rs3134069) were genotyped by TaqMan assays in 2027 Spanish patients with rheumatoid arthritis. Anti-cyclic citrullinated peptide (anti-CCP) antibody testing was positive in 997 of 1714 tested. Also, 18.3% of the whole series had experienced cardiovascular events, including 5.4% with cerebrovascular accidents. The relationship between *OPG* variants and cardiovascular events was assessed using Cox regression.

**Results:**

No association between *OPG* gene variants and cardiovascular disease was observed in the whole group of rheumatoid arthritis patients or in anti-CCP positive patients. Nevertheless, a protective effect of CGA haplotype on the risk of cardiovascular disease in general, and specifically in the risk of cerebrovascular complications after adjusting for sex, age at disease diagnosis and traditional cardiovascular risk factors was disclosed in anti-CCP negative patients (HR = 0.54; 95%CI: 0.31–0.95; p = 0.032 and HR = 0.17; 95%CI: 0.04–0.78; p = 0.022, respectively).

**Conclusion:**

Our results indicate a protective effect of the *OPG* CGA haplotype on cardiovascular risk, mainly due to a protective effect against cerebrovascular events in anti-CCP negative rheumatoid arthritis patients.

## Introduction

Rheumatoid arthritis (RA) is a chronic inflammatory rheumatic disease associated with high incidence of cardiovascular (CV) morbidity and mortality compared to the general population [1]–[4], similarly to what occurs in type 2 diabetes [5], [6]. Specifically, it has been shown a high incidence of coronary heart disease and a high rate of CV events in RA [7], [8] due to accelerated atherosclerosis [9]. Besides traditional CV risk factors [4], [10] and the magnitude and severity of the chronic inflammatory response [8], genetic factors located inside [8] and outside the Human Leukocyte Antigen (HLA) region [11], [12] play a pivotal role in the development of atherogenesis in RA [13]–[15].

Osteoprotegerin (OPG) belongs to the TNF receptor super-family and is implicated in bone remodeling and in the atherosclerotic process. This molecule acts as a decoy receptor for the receptor activator of nuclear factor-κB ligand (RANKL), inhibiting binding of RANKL to its receptor, RANK [16], [17]. Binding of RANKL to OPG inhibits osteoclastogenesis, although it is also well known that both molecules are involved in vascular wall mineralization [16]. Additionally, OPG acts as a soluble neutralizing receptor of TNF-related apoptosis-inducing ligand (TRAIL), an anti-inflammatory molecule with anti-atherosclerotic properties [18]–[20]. Despite having a paradoxically protective effect on vascular calcification [21], [22], OPG has been associated with increased risk of atherosclerotic disease in the general population [23].

The human *OPG* gene (also called *TNFRSF11B*) is located on chromosome 8q24. This gene is affected by genetic polymorphisms with functional consequences on CV disease and bone metabolism [24], [25]. Recently, several groups have reported that a single-nucleotide OPG polymorphism (SNP) located in the 5′ UTR region (rs2073617), as well as one in exon 1 (rs2073618) and another in the promoter region (rs3134069) were associated with atherosclerosis and risk of cerebrovascular disease in non-rheumatic individuals [26], [27].

Considering the functional involvement of the above mentioned *OPG* polymorphisms in the CV disease, in the present study we aimed to analyze the potential association of these gene variations on the risk of developing CV disease in a large and well-characterized cohort of patients with RA, also evaluating their combined effect on this risk.

## Materials and Methods

### Patients and Study Protocol

**Figure 1:**
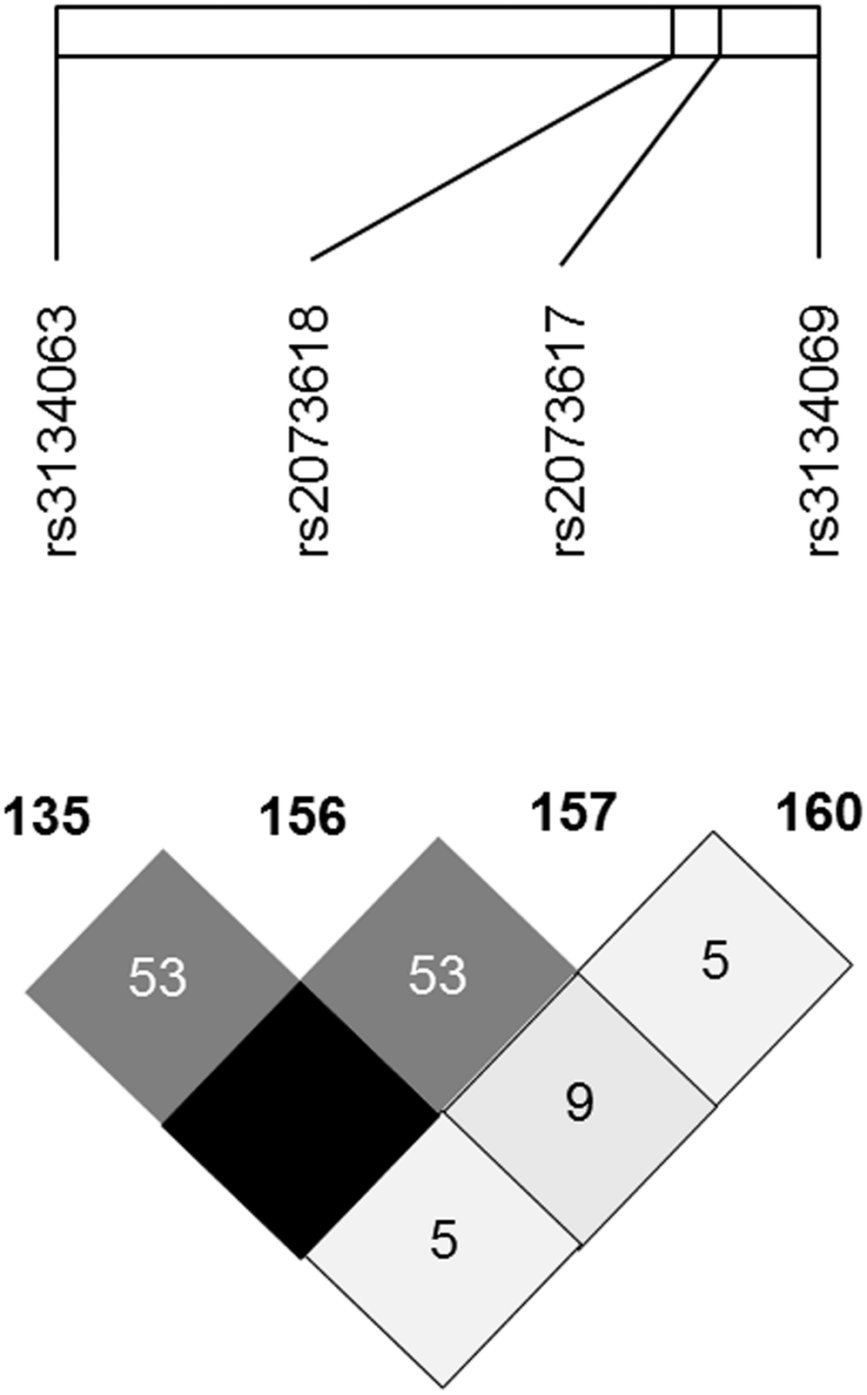
Linkage disequilibrium (LD) pattern of the OPG polymorphisms analyzed in our study (in European population). Data obtained by HapMap Project phase I, II and III and HAPLOVIEW (v.4.2) software. The LD between the OPG polymorphisms studied is shown in a scale from minimum (white) to maximum (black).

A set of 2027 Spanish patients with RA were included in the present study. Blood samples were obtained from patients recruited from Hospital Lucus Augusti (Lugo), Hospital Marqués de Valdecilla (Santander), Hospital de Bellvitge (Barcelona), and Hospital Clínico San Carlos, Hospital La Paz, Hospital La Princesa, Hospital Gregorio Marañón and Hospital 12 de Octubre (Madrid). A subject’s written consent was obtained according to the declaration of Helsinki, and the study was approved by the Ethics Committee of Galicia for Hospital Lucus Augusti, of Cantabria for Hospital Universitario Marqués de Valdecilla, of Cataluña for Hospital de Bellvitge and of Madrid for Hospital Clínico San Carlos, Hospital La Paz, Hospital La Princesa, Hospital Gregorio Marañón and Hospital 12 de Octubre. All the patients fulfilled the 1987 American College of Rheumatology (ACR) and also the 2010 classification criteria for RA [28], [29]. In all the cases, the samples were assessed for *OPG* rs2073618 and rs3134069 polymorphisms. Additionally, rs2073617 polymorphism was assessed with a pre-designed Taqman probe for the rs3134063 polymorphism, which is in complete linkage disequilibrium with rs2073617 (r^2^ = 1, http://hapmap.ncbi.nlm.nih.gov/). The linkage disequilibrium (LD) pattern of the *OPG* polymorphisms analyzed in our study obtained by HapMap Project phase I, II and III (in the European population) and HAPLOVIEW (v.4.2) software is displayed in **Figure 1**.

Information on the main demographic data, clinical characteristics, CV risk factors and CV events of patients enrolled in the study is shown in **Table 1**. Anti-cyclic citrullinated peptide (anti-CCP) antibody testing were positive in 997 (58.2%) of 1714 RA patients in whom this result was available. Three hundred and seventy (18.3%) of these 2027 patients had experienced CV events. One-hundred and nine (5.4%) of the 2027 patients had suffered cerebrovascular accidents. Definitions of CV events and traditional CV risk factors were established as previously described [8], [30].

**Table 1:** Demographic and clinical characteristics of the Spanish patients with RA included in the study.

Clinical Feature		% (n/N)
Patients		2027
Main characteristics	Age at the time of disease onset (years, mean ± SD)	51.2±14.9
	Follow-up (years, mean ± SD)	11.7±8.4
	Female gender	75.3 (1527/2027)
	Rheumatoid factor positive*	68.6 (1344/1958)
	Anti-CCP antibodies positive	58.2 (997/1714)
	Shared epitope positive	63.7 (726/1139)
Cardiovascular risk factors	Hypertension	38.2 (763/1996)
	Diabetes mellitus	12.3 (246/1995)
	Dyslipidemia	35.9 (713/1984)
	Obesity	18.8 (345/1840)
	Smoking habit	24.9 (491/1974)
Patients with cardiovascular events		18.3 (370/2027)
	Ischemic heart disease	8.9 (180/2020)
	Heart failure	5.6 (113/2027)
	Cerebrovascular accident	5.4 (109/2027)
	Peripheral arteriopathy	2.6 (52/2025)

CCP: cyclic citrullinated protein/peptide antibodies; RA: rheumatoid arthritis; SD: standard deviation.

*At least two determinations at different times were required.

### Genotyping

DNA from patients was obtained from peripheral blood using standard methods. The *OPG* rs3134063, rs2073618 and rs3134069 polymorphisms were genotyped with TaqMan SNP pre-designed genotyping assays (C__32324439_10, C___1971047_1_, and C__27464534_10, respectively) in a 7900 HT real-time polymerase chain reaction (PCR) system, according to the conditions recommended by the manufacturer (Applied Biosystem, Foster City, CA, USA). Negative controls and duplicate samples were included to check the accuracy of genotyping.

### Statistical analysis

The genotype data were checked for deviation from Hardy-Weinberg equilibrium (HWE) using http://ihg.gsf.de/cgi-bin/hw/hwa1.pl.

The relationship between alleles, genotypes and haplotypes, and CV events that occurred in the follow-up was tested using Cox regression adjusting for sex, age at RA diagnosis and traditional CV risk factors. For that purpose, we used the most frequent allele, genotype and haplotype as reference; the end of follow-up was the first date among the end of the study, date of death or date of CV event. Follow-up time was estimated as the difference between the RA diagnosis date and the end of follow-up. Patients without CV events in the follow-up time or dying by any non-CV cause were considered as censored. Results were expressed as hazard ratios (HR) with 95% confidence interval (CI). Statistical significance was defined as p≤0.05, and all analyses were performed using STATA statistical software 12/SE (Stata Corp., College Station, TX, USA).

## Results

*OPG* rs3134063, rs2073618 and rs3134069 genotype distribution was in Hardy-Weinberg equilibrium (p>0.05). Genotyping success was greater than 96% in all the cases. Genotype and allele frequencies of the *OPG* rs3134063, rs2073618 and rs3134069 polymorphisms were in agreement with the data of the HapMap project (http://hapmap.ncbi.nlm.nih.gov/).

There was no association between *OPG* gene variants and CV disease when all the RA patients were assessed as a whole. It was also the case for the group of anti-CCP positive patients (data not shown). Nevertheless, an association between *OPG* gene polymorphisms and CV disease was observed in those who were anti-CCP negative (**Table 2**). In this regard, even if no association was observed between allelic and genotypic *OPG* variants and CV disease (not shown), in the haplotype analysis, a protective effect of the CGA haplotype on the risk of CV disease after adjusting for sex, age at RA diagnosis and traditional CV risk factors was disclosed in the group of anti-CCP negative RA patients (HR = 0.54; 95% CI: 0.31–0.95; p = 0.032) (**Table 2**).

**Table 2:** Results of haplotype analysis in anti-CCP negative RA patients in association with CV events.

*Variable*		*HR (95% CI)*	p
	**Age at RA diagnosis** **(by each year)**	**1.08 (1.06–1.10)**	**<0.001**
	Hypertension	0.98 (0.63–1.51)	0.929
	**Diabetes mellitus**	**1.58 (1.01–2.45)**	**0.043**
	Obesity	1.22 (0.78–1.89)	0.390
	Dyslipidemia	1.27 (0.84–1.93)	0.254
	Smoking	1.12 (0.85–1.48)	0.417
***Haplotypes (rs3134063, rs2073618, rs3134069)***		***HR (95% CI)*** *	***p*** *
	TCA	1 (reference)	-
	TGA	0.94 (0.55–1.60)	0.825
	CCA	1.09 (0.68–1.76)	0.724
	**CGA**	**0.54 (0.31–0.95)**	**0.032**

CCP: cyclic citrullinated protein/peptide antibodies; CI: confidence interval; CV: cardiovascular; HR: hazard ratios; RA: rheumatoid arthritis.

*Adjusted for sex, age at RA diagnosis and traditional CV risk factors (hypertension, diabetes mellitus, dyslipidemia, obesity, and smoking habit).

In a further step, we aimed to determine the type of CV event associated with *OPG* gene variants in the group of anti-CCP negative patients. Whereas no association was observed with ischemic heart disease (data not shown), we found an association of cerebrovascular complications with rs2073618 *OPG* polymorphism in this group of patients. In this respect, the risk of cerebrovascular complications was statistically decreased in the group of anti-CCP negative patients who carried the *OPG* rs2073618 GG genotype after adjusting the results for potential confounder factors (HR = 0.17; 95% CI: 0.03–0.89; p = 0.035) (**Table 3**). In accordance with these results, a protective effect of the CGA haplotype on the risk of cerebrovascular events after adjusting for sex, age at RA diagnosis and traditional CV risk factors was observed in the anti-CCP negative RA patients (HR = 0.17; 95% CI: 0.04–0.78; p = 0.022) (**Table 4**).

**Table 3:** Association between *OPG* polymorphisms and the risk to develop cerebrovascular events in anti-CCP negative RA patients.

*SNP*	*Genotype/Allele*	*HR (95%CI)*	*p* *
rs3134063	TT	1 (reference)	-
	CT	0.61 (0.21–1.76)	0.362
	CC	0.14 (0.02–1.23)	0.077
	T	1 (reference)	-
	C	0.52 (0.26–1.06)	0.072
rs2073618	CC	1 (reference)	-
	CG	0.60 (0.20–1.82)	0.368
	**GG**	**0.17 (0.03–0.89)**	**0.035**
	C	1 (reference)	-
	**G**	**0.45 (0.22–0.91)**	**0.025**
rs3134069	AA	1 (reference)	-
	AC	1.20 (0.26–5.64)	0.814
	CC	-	-
	A	1 (reference)	-
	C	1.18 (0.27–5.09)	0.823

CCP: cyclic citrullinated protein/peptide antibodies; CI: confidence interval; HR: hazard ratios; RA: rheumatoid arthritis.

*Adjusted for sex, age at RA diagnosis and traditional CV risk factors (hypertension, diabetes mellitus, dyslipidemia, obesity, and smoking habit).

**Table 4:** Results of haplotype analysis in anti-CCP negative RA patients in association with cerebrovascular events.

*Variable*		*HR (95% CI)*	p
	**Age at RA diagnosis** **(by each year)**	**1.09 (1.05–1.13)**	**<0.001**
	Hypertension	1.13 (0.51–2.48)	0.769
	**Diabetes mellitus**	**3.71 (1.78–7.70)**	**<0.001**
	Obesity	1.28 (0.58–2.83)	0.547
	Dyslipidemia	0.80 (0.39–1.66)	0.556
	Smoking	1.13 (0.68–1.88)	0.632
***Haplotypes (rs3134063, rs2073618, rs3134069)***		***HR (95% CI)*** *	***p*** *
	TCA	1 (reference)	-
	TGA	1.38 (0.56–3.42)	0.487
	CCA	1.16 (0.49–2.76)	0.740
	**CGA**	**0.17 (0.04–0.78)**	**0.022**

CCP: cyclic citrullinated protein/peptide antibodies; CI: confidence interval; CV: cardiovascular; HR: hazard ratios; RA: rheumatoid arthritis.

*Adjusted for sex, age at RA diagnosis and traditional CV risk factors (hypertension, diabetes mellitus, dyslipidemia, obesity, and smoking habit).

## Discussion

RA is associated with increased morbidity and mortality attributable to accelerated atherosclerosis and CV events [7], [9], in a similar fashion to what is observed in other autoimmune diseases such as diabetes [6]. Therefore, the search of new genetic markers which could help physicians to stratify RA patients according to their CV risk, leading thus to an improved and more personalized treatment, has become a main goal for several groups of research.

OPG has been proposed as a potential biomarker of CV risk since increased levels of this protein have been associated with CV disease [23]. In this regard, OPG levels were associated with biomarkers of endothelial activation (intercellular adhesion molecule-1), carotid intima-media wall thickness and carotid plaques in RA patients with severe disease [31]. In keeping with these results, an independent correlation of OPG levels with asymmetric dimethylarginine (ADMA), another biomarker of endothelial cell activation, has been disclosed in ankylosing spondylitis patients undergoing anti-TNF-α therapy [32]. Increasing concentrations of OPG have also been associated with the severity of CV complications in diabetic patients [33]–[37], reinforcing the idea that OPG could be used as a prognostic factor of CV disease in the clinic.

Several groups have described sequence variations in the gene that codifies OPG [24]–[26]. As elegantly proposed by Soufi et al., variations in the different regions of the *OPG* gene could combine to affect its transcription, intracellular trafficking or secretion [24]. To the best of our knowledge, our study constitutes the first attempt to assess the potential effect of rs3134063, rs2073618 and rs3134069 on the risk to develop CV disease in a large and well-characterized cohort of RA patients.

When we studied the influence of the different *OPG* polymorphisms on the risk of CV disease, we found a protective effect of the CGA haplotype on the risk of CV events in the subgroup of RA patients who were anti-CCP negative. Interestingly, further analyses disclosed that this protective effect was specifically focused on the risk of developing cerebrovascular accidents. In this regard, anti-CCP negative RA patients who carried an *OPG* rs20730618 GG genotype had a lower risk of developing cerebrovascular complications. Additionally, when we combined the different genetic variants to create haplotypes, our results also revealed a protective effect of the CGA haplotype (that carries the G allele of the *OPG* rs2073618) against the risk of cerebrovascular events in the subgroup of anti-CCP negative RA patients. These results are in line with those obtained by Biscetti et al., who found a synergistic effect of rs3134069, rs2073617 and rs2073618 associated with cerebral ischemic events in a cohort of diabetic patients [27]. Likewise, our results are in accordance with those obtained by Mankoč Ramuš et al., who also found an association between a combination of two of the *OPG* gene variants studied by our group and diabetic retinopathy [38].

Interestingly, the protective effect of the *OPG* CGA haplotype was found in anti-CCP negative but not anti-CCP positive RA patients. This could be explained by the fact that anti-CCP positive and anti-CCP negative RA are considered different disease entities [39]. Several pieces of evidence disclose that anti-CCP antibodies are markers of severe disease, and that risk factors such as HLA class II alleles associate with anti-CCP status and a more severe disease course [40]. In this regard, anti-CCP positive RA patients display a more severe radiological destruction and elevated DAS28 and CRP values than anti-CCP negative patients [39], [41].

Taken all these considerations together, our results and those previously mentioned support the idea that, in an attempt to establish the potential association between multiple genetic markers in a chromosomal region and traits of interest in complex diseases, haplotype analyses appear to provide more useful information than the separate assessment of individual gene variants [27], [38], [42]–[45]. Hence, combination analyses often help to disclose hidden signals and may tag other regional polymorphic sites.

## Conclusion

Our results indicate a protective effect of the *OPG* CGA haplotype on CV risk, mainly due to a protective effect against cerebrovascular events in anti-CCP negative RA patients.

## References

[1] Avina-Zubieta JA, Thomas J, Sadatsafavi M, Lehman AJ, Lacaille D (2012) Risk of incident cardiovascular events in patients with rheumatoid arthritis: a meta-analysis of observational studies. Ann Rheum Dis 71: 1524–1529. 22425941 10.1136/annrheumdis-2011-200726

[2] Goodson N, Marks J, Lunt M, Symmons D (2005) Cardiovascular admissions and mortality in an inception cohort of patients with rheumatoid arthritis with onset in the 1980s and 1990s. Ann Rheum Dis 64: 1595–1601. 15843450 10.1136/ard.2004.034777 PMC1755282

[3] Solomon DH, Karlson EW, Rimm EB, Cannuscio CC, Mandl LA, et al (2003) Cardiovascular morbidity and mortality in women diagnosed with rheumatoid arthritis. Circulation 107: 1303–1307. 12628952 10.1161/01.cir.0000054612.26458.b2

[4] Del Rincon ID, Williams K, Stern MP, Freeman GL, Escalante A (2001) High incidence of cardiovascular events in a rheumatoid arthritis cohort not explained by traditional cardiac risk factors. Arthritis Rheum 44: 2737–2745. 11762933 10.1002/1529-0131(200112)44:12<2737::AID-ART460>3.0.CO;2-%23

[5] Peters MJ, van Halm VP, Voskuyl AE, Smulders YM, Boers M, et al (2009) Does rheumatoid arthritis equal diabetes mellitus as an independent risk factor for cardiovascular disease? A prospective study. Arthritis Rheum 61: 1571–1579. 19877093 10.1002/art.24836

[6] van Halm VP, Peters MJ, Voskuyl AE, Boers M, Lems WF, et al (2009) Rheumatoid arthritis versus diabetes as a risk factor for cardiovascular disease: a cross-sectional study, the CARRE Investigation. Ann Rheum Dis 68: 1395–1400. 18697775 10.1136/ard.2008.094151

[7] Wolfe F, Freundlich B, Straus WL (2003) Increase in cardiovascular and cerebrovascular disease prevalence in rheumatoid arthritis. J Rheumatol 30: 36–40. 12508387

[8] Gonzalez-Gay MA, Gonzalez-Juanatey C, Lopez-Diaz MJ, Piñeiro A, Garcia-Porrua C, et al (2007) HLA-DRB1 and persistent chronic inflammation contribute to cardiovascular events and cardiovascular mortality in patients with rheumatoid arthritis. Arthritis Rheum 57: 125–132. 17266100 10.1002/art.22482

[9] Gonzalez-Gay MA, Gonzalez-Juanatey C, Martin J (2005) Rheumatoid arthritis: a disease associated with accelerated atherogenesis. Semin Arthritis Rheum 35: 8–17. 16084219 10.1016/j.semarthrit.2005.03.004

[10] Dessein PH, Joffe BI, Veller MG, Stevens BA, Tobias M, et al (2005) Traditional and nontraditional cardiovascular risk factors are associated with atherosclerosis in rheumatoid arthritis. J Rheumatol 32: 435–442. 15742434

[11] García-Bermúdez M, López-Mejías R, Genre F, Castañeda S, González-Juanatey C, et al (2013) SMAD3 rs17228212 gene polymorphism is associated with reduced risk to cerebrovascular accidents and subclinical atherosclerosis in anti-CCP negative Spanish rheumatoid arthritis patients. PLoS One 8: e77695. 24204921 10.1371/journal.pone.0077695 PMC3804609

[12] López-Mejías R, García-Bermúdez M, González-Juanatey C, Castañeda S, Miranda-Filloy JA, et al (2012) NFKB1-94ATTG ins/del polymorphism (rs28362491) is associated with cardiovascular disease in patients with rheumatoid arthritis. Atherosclerosis 224: 426–429. 22742859 10.1016/j.atherosclerosis.2012.06.008

[13] Gonzalez-Gay MA, Gonzalez-Juanatey C, Piñeiro A, Garcia-Porrua C, Testa A, et al (2005) High-grade C-reactive protein elevation correlates with accelerated atherogenesis in patients with rheumatoid arthritis. J Rheumatol 32: 1219–1223. 15996055

[14] Del Rincón I, Williams K, Stern MP, Freeman GL, O’Leary DH, et al (2003) Association between carotid atherosclerosis and markers of inflammation in rheumatoid arthritis patients and healthy subjects. Arthritis Rheum 48: 1833–1840. 12847676 10.1002/art.11078

[15] Rodríguez-Rodríguez L, López-Mejías R, García-Bermúdez M, González-Juanatey C, González-Gay MA, et al (2012) Genetic markers of cardiovascular disease in rheumatoid arthritis. Mediators Inflamm 2012: 574817. 22927710 10.1155/2012/574817 PMC3419432

[16] Hofbauer LC, Schoppet M (2004) Clinical implications of the osteoprotegerin/RANKL/RANK system for bone and vascular diseases. JAMA 292: 490–495. 15280347 10.1001/jama.292.4.490

[17] Van Campenhout A, Golledge J (2009) Osteoprotegerin, vascular calcification and atherosclerosis. Atherosclerosis 204: 321–329. 19007931 10.1016/j.atherosclerosis.2008.09.033 PMC2729052

[18] Secchiero P, Corallini F, Beltrami AP, Ceconi C, Bonasia V, et al (2010) An imbalanced OPG/TRAIL ratio is associated to severe acute myocardial infarction. Atherosclerosis 210: 274–277. 20015493 10.1016/j.atherosclerosis.2009.11.005

[19] Secchiero P, Rimondi E, di Iasio MG, Agnoletto C, Melloni E, et al (2013) C-reactive protein downregulates TRAIL expression in human peripheral monocytes via an Egr-1-dependent pathway. Clin Cancer Res 19: 1949–1959. 23468057 10.1158/1078-0432.CCR-12-3027

[20] Di Bartolo BA, Cartland SP, Harith HH, Bobryshev YV, Schoppet M, et al (2013) TRAIL-deficiency accelerates vascular calcification in atherosclerosis via modulation of RANKL. PLoS One 8: e74211. 24040204 10.1371/journal.pone.0074211 PMC3764101

[21] Callegari A, Coons ML, Ricks JL, Yang HL, Gross TS, et al (2013) Bone marrow- or vessel wall-derived osteoprotegerin is sufficient to reduce atherosclerotic lesion size and vascular calcification. Arterioscler Thromb Vasc Biol 33: 2491–2500. 23990207 10.1161/ATVBAHA.113.301755

[22] Morony S, Tintut Y, Zhang Z, Cattley RC, Van G, et al (2008) Osteoprotegerin inhibits vascular calcification without affecting atherosclerosis in ldlr (−/−) mice. Circulation 117: 411–420. 18172035 10.1161/CIRCULATIONAHA.107.707380 PMC2680735

[23] Kiechl S, Schett G, Wenning G, Redlich K, Oberhollenzer M, et al (2004) Osteoprotegerin is a risk factor for progressive atherosclerosis and cardiovascular disease. Circulation 109: 2175–2180. 15117849 10.1161/01.CIR.0000127957.43874.BB

[24] Soufi M, Schoppet M, Sattler AM, Herzum M, Maisch B, et al (2004) Osteoprotegerin gene polymorphisms in men with coronary artery disease. J Clin Endocrinol Metab 89: 3764–3768. 15292302 10.1210/jc.2003-032054

[25] Roshandel D, Holliday KL, Pye SR, Ward KA, Boonen S, et al (2011) Influence of polymorphisms in the RANKL/RANK/OPG signaling pathway on volumetric bone mineral density and bone geometry at the forearm in men. Calcif Tissue Int 89: 446–455. 21964949 10.1007/s00223-011-9532-y PMC3215872

[26] Straface G, Biscetti F, Pitocco D, Bertoletti G, Misuraca M, et al (2011) Assessment of the genetic effects of polymorphisms in the osteoprotegerin gene, TNFRSF11B, on serum osteoprotegerin levels and carotid plaque vulnerability. Stroke 42: 3022–3028. 21903966 10.1161/STROKEAHA.111.619288

[27] Biscetti F, Straface G, Giovannini S, Santoliquido A, Angelini F, et al (2013) Association between TNFRSF11B gene polymorphisms and history of ischemic stroke in Italian diabetic patients. Hum Genet 132: 49–55. 22965192 10.1007/s00439-012-1224-9

[28] Arnett FC, Edworthy SM, Bloch DA, McShane DJ, Fries JF, et al (1988) The American Rheumatism Association 1987 revised criteria for the classification of rheumatoid arthritis. Arthritis Rheum 31: 315–324. 3358796 10.1002/art.1780310302

[29] Aletaha D, Neogi T, Silman AJ, Funovits J, Felson DT, et al (2010) 2010 Rheumatoid arthritis classification criteria: an American College of Rheumatology/European League Against Rheumatism collaborative initiative. Arthritis Rheum 62: 2569–2581. 20872595 10.1002/art.27584

[30] Gonzalez-Juanatey C, Llorca J, Martin J, Gonzalez-Gay MA (2009) Carotid intima-media thickness predicts the development of cardiovascular events in patients with rheumatoid arthritis. Semin Arthritis Rheum 38: 366–371. 18336869 10.1016/j.semarthrit.2008.01.012

[31] Dessein PH, López-Mejias R, González-Juanatey C, Genre F, Miranda-Filloy JA, et al (2014) Independent Relationship of Osteoprotegerin Concentrations with Endothelial Activation and Carotid Atherosclerosis in Patients with Severe Rheumatoid Arthritis. J Rheumatol 41: 429–436. 24488413 10.3899/jrheum.131037

[32] Genre F, López-Mejías R, Miranda-Filloy JA, Ubilla B, Carnero-López B, et al. (2014) Osteoprotegerin correlates with disease activity and endothelial activation in non-diabetic ankylosing spondylitis patients undergoing TNF-α antagonist therapy. Clin Exp Rheumatol: In press. 25190453

[33] Chen WJ, Rijzewijk LJ, van der Meer RW, Heymans MW, van Duinkerken E, et al (2011) Association of plasma osteoprotegerin and adiponectin with arterial function, cardiac function and metabolism in asymptomatic type 2 diabetic men. Cardiovasc Diabetol 10: 67. 21771299 10.1186/1475-2840-10-67 PMC3157422

[34] Jorsal A, Tarnow L, Flyvbjerg A, Parving HH, Rossing P, et al (2008) Plasma osteoprotegerin levels predict cardiovascular and all-cause mortality and deterioration of kidney function in type 1 diabetic patients with nephropathy. Diabetologia 51: 2100–2107. 18719882 10.1007/s00125-008-1123-8

[35] Anand DV, Lahiri A, Lim E, Hopkins D, Corder R (2006) The relationship between plasma osteoprotegerin levels and coronary artery calcification in uncomplicated type 2 diabetic subjects. J Am Coll Cardiol 47: 1850–1857. 16682312 10.1016/j.jacc.2005.12.054

[36] Rasmussen LM, Tarnow L, Hansen TK, Parving HH, Flyvbjerg A (2006) Plasma osteoprotegerin levels are associated with glycaemic status, systolic blood pressure, kidney function and cardiovascular morbidity in type 1 diabetic patients. Eur J Endocrinol 154: 75–81. 16381994 10.1530/eje.1.02049

[37] Bjerre M (2013) Osteoprotegerin (OPG) as a biomarker for diabetic cardiovascular complications. Springerplus 2: 658. 24349960 10.1186/2193-1801-2-658 PMC3863400

[38] Mankoč Ramuš S, Kumše T, Globočnik Petrovič M, Petrovič D, Cilenšek I (2013) SNP rs2073618 of the osteoprotegerin gene is associated with diabetic retinopathy in Slovenian patients with type 2 diabetes. Biomed Res Int 2013: 364073. 24228244 10.1155/2013/364073 PMC3817801

[39] van der Helm-van Mil AH, Verpoort KN, Breedveld FC, Toes RE, Huizinga TW (2005) Antibodies to citrullinated proteins and differences in clinical progression of rheumatoid arthritis. Arthritis Res Ther 7(5): R949–958. 16207336 10.1186/ar1767 PMC1257421

[40] van Gaalen FA, van Aken J, Huizinga TW, Schreuder GM, Breedveld FC, et al (2004) Association between HLA class II genes and autoantibodies to cyclic citrullinated peptides (CCPs) influences the severity of rheumatoid arthritis. Arthritis Rheum 50(7): 2113–2121. 15248208 10.1002/art.20316

[41] del Val del Amo N, Ibanez Bosch R, Fito Manteca C, Gutierrez Polo R, Loza Cortina E (2006) Anti-cyclic citrullinated peptide antibody in rheumatoid arthritis: relation with disease aggressiveness. Clin Exp Rheumatol 24(3): 281–286. 16870095

[42] Zhao H, Pfeiffer R, Gail MH (2003) Haplotype analysis in population genetics and association studies. Pharmacogenomics 4: 171–178. 12605551 10.1517/phgs.4.2.171.22636

[43] Morris RW, Kaplan NL (2002) On the advantage of haplotype analysis in the presence of multiple disease susceptibility alleles. Genet Epidemiol 23: 221–233. 12384975 10.1002/gepi.10200

[44] Martin ER, Lai EH, Gilbert JR, Rogala AR, Afshari AJ, et al (2000) SNPing away at complex diseases: analysis of single-nucleotide polymorphisms around APOE in Alzheimer disease. Am J Hum Genet 67: 383–394. 10869235 10.1086/303003 PMC1287185

[45] Drysdale CM, McGraw DW, Stack CB, Stephens JC, Judson RS, et al (2000) Complex promoter and coding region beta 2-adrenergic receptor haplotypes alter receptor expression and predict in vivo responsiveness. Proc Natl Acad Sci USA 97: 10483–10488. 10984540 10.1073/pnas.97.19.10483 PMC27050

